# The Impact of Harm Review Service on Patients Awaiting Elective Orthopaedic Foot and Ankle Surgery for More Than 52 Weeks

**DOI:** 10.7759/cureus.23444

**Published:** 2022-03-24

**Authors:** Siddharth Virani, Oubida Asaad, Omkaar Divekar, Crispin Southgate, Baljinder S Dhinsa

**Affiliations:** 1 Trauma and Orthopaedics, William Harvey Hospital, Ashford, GBR

**Keywords:** harm review clinic during covid-19, elective foot and ankle surgeries, foot and ankle 52 weeks wait, efficacy of harm review clinic, harm review service impact

## Abstract

Background and objective

There has been a significant increase in waiting times for elective surgical procedures in orthopaedic surgery as a result of the coronavirus disease 2019 (COVID-19) pandemic. As per the hospital policy, patients awaiting elective surgery for more than 52 weeks were offered a consultant-led harm review. The aim of this study was to objectively assess the impact of this service on the field of foot and ankle surgery.

Materials and methods

The data from harm review clinics at a District General Hospital related to patients waiting to undergo elective foot and ankle procedures in the year 2021 (wait time of more than 52 weeks) were assessed. Clinical data points like change in diagnosis, need for further investigations, and patients being taken off the waiting list were reviewed. The effect of the waiting time on patients’ mental health and their perception of the service was assessed as well.

Results

A total of 72 patients awaiting foot and ankle procedures for more than 52 weeks were assessed as a part of the harm review service. It was noted that 25% of patients found that their symptoms had worsened while 66.1% perceived them to be unchanged. Twelve patients (16.9%) were sent for updated investigations. Twenty-one patients (29.5%) were taken off the waiting lists for various reasons with the most common one being other pressing health concerns; 9% of patients affirmed that the wait for surgery had a significant negative impact on their mental health.

Conclusion

This study concludes that the harm review service is a useful programme as it helps guide changes in the diagnosis and clinical picture. The service is found to be valuable by most patients, and its impact on the service specialities and multiple centres could be further assessed to draw broad conclusions.

## Introduction

A significant drop in the elective surgical output across specialities was observed during the year 2020 due to resources being diverted to tackling the coronavirus disease 2019 (COVID-19) crisis [1]. This has led to a significant number of patients being forced to wait an inordinate amount of time to receive elective treatments in trauma and orthopaedic surgery. A statement by the British Orthopaedic Association in January 2021 noted that 67,137 orthopaedic patients had been waiting to receive elective surgical care for over a year compared to just 436 in January 2020 [2].

The term harm implies impairment of a structure or function of the body and it could be physical, psychological, or social [3]. In the context of patients waiting for an operation, the purpose of clinical harm review is to identify and correct any harm arising from the prolonged wait time. Additionally, the harm review process also aims to learn lessons, draw conclusions, and mitigate further recurrences of the conditions [3].

The aim of this study was to objectively assess the impact of the harm review service on the field of elective foot and ankle surgery. The use of this programme has become widespread due to the repeated postponements of elective surgeries during the ongoing COVID-19 pandemic. Our objectives were to quantify the patients’ experience of being on a waiting list for prolonged periods of time and to judge its impact on their perception of their morbidity and mental health. We also aimed to assess the clinician’s perspective of the efficacy of the harm review service.

## Materials and methods

All patients waiting for elective surgeries for more than 52 weeks were offered a consultant-led harm review virtual clinic as per the Trust policy. In this study, the harm review service for patients awaiting elective surgery by the foot and ankle team was assessed. These review clinics were conducted via telephone by consultant foot and ankle surgeons (C.S. and B.D.) to ascertain harm among patients waiting more than 52 weeks. These clinics were held in the period of January-March 2021.

The outcomes of these clinics were then independently assessed by two observers to ensure reliability. Conflicting data points were then assessed by a third independent observer and were also verified by the consultant. The clinic letters were used as a surrogate for the clinical consultation. Patient demographic factors, diagnoses, and the procedures listed were recorded. From the patients’ perspective, it was assessed if the perceived symptoms had become better, remained unchanged, or become worse. It was also assessed subjectively if patients’ mental health had been affected in the form of anxiety, depression, or apprehension. Finally, an assessment as to whether the patients considered the consultation to be valuable was also performed.

From the perspective of the clinician, it was examined if the clinical diagnosis had changed over the waiting period. Further, the number of patients needing fresh radiological investigations was also documented. Also, the number of patients needing a face-to-face review in the clinic for clinical examination was assessed. The documentation was also studied to see if patients were taken off the waiting list and the reasons for the same. Finally, a note was made if the consultant had documented if any harm had been done as a result of the waiting time.

The data was organised and tabulated in a Microsoft Excel sheet (Microsoft Corp, Redmond, WA). An objective analysis of the results was performed to quantify the impact of the service.

## Results

Seven clinics assessing a total of 72 patients awaiting an elective foot and ankle procedure were conducted in the above-mentioned time period. One patient died from an unrelated medical condition, and hence 71 patients were ultimately assessed. The mean age of the patients was 62.9 years. There were 44 females while the rest were males.

**Figure 1:**
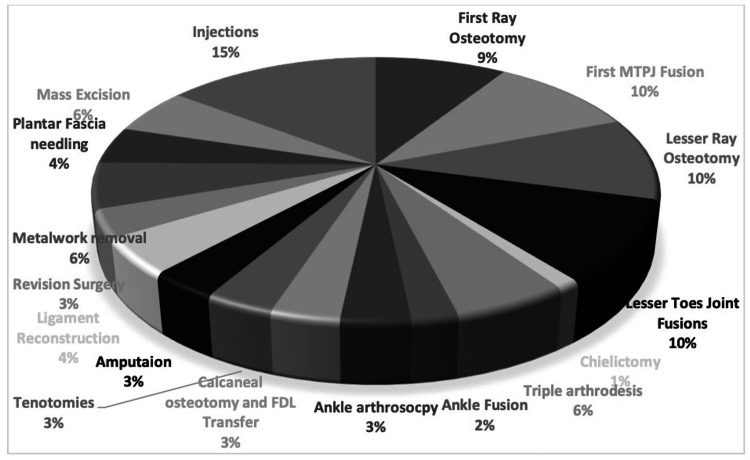
Distribution of diagnoses of patients reviewed under the harm evaluation service FDL: flexor digitorum longus; MTPJ: metatarsophalangeal joint

Apart from targeted injections under fluoroscopy, the most common procedures for which the patients were listed included first metatarsophalangeal joint (MTPJ) fusion (10%), first ray and lesser ray osteotomies (9% and 10% respectively), and lesser toe fusions (10%). Other common procedures included triple arthrodesis, calcaneal osteotomies, ligament reconstructions, ankle fusions, and arthroscopies; 6% of the patients were awaiting metalwork removal while 3% were waiting for a revision procedure (Figure 1).

**Figure 2:**
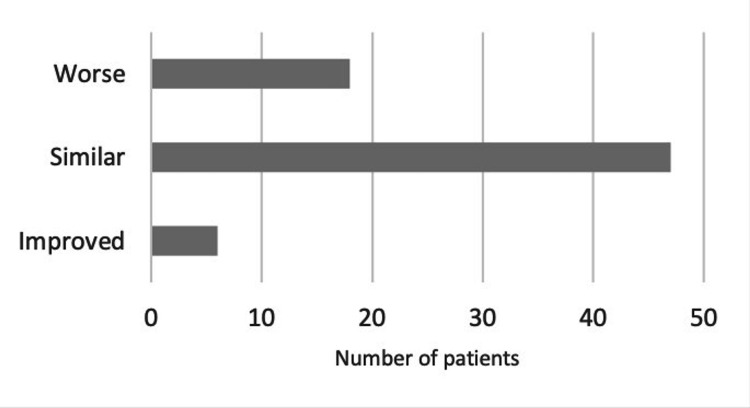
Patients' perception of symptoms while waiting for foot and ankle surgery

Forty-seven patients (66.1%) perceived that their clinical symptoms remained unchanged despite waiting for more than a year. Six patients reported an improvement in their clinical picture, while 18 patients (25%) believed their symptoms had worsened. Seventy patients (98.5%) appreciated the consultation and found it valuable (Figure 2).

**Figure 3:**
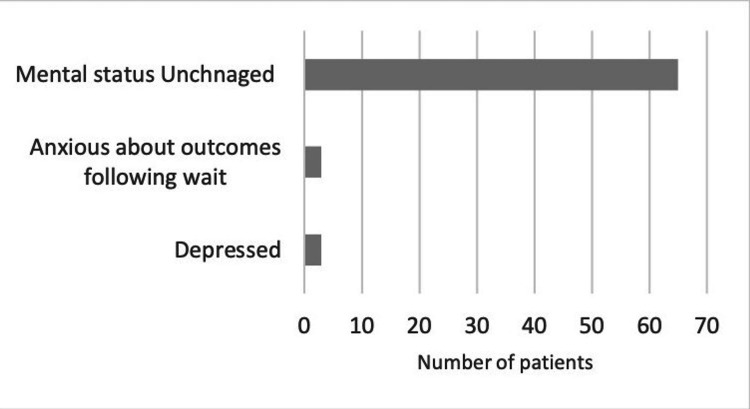
Impact of the wait for elective surgery on patients' mental health

About 9% of patients felt that their mental health was affected in the form of anxiety or depression due to the prolonged waiting for the procedure (Figure 3).

**Figure 4:**
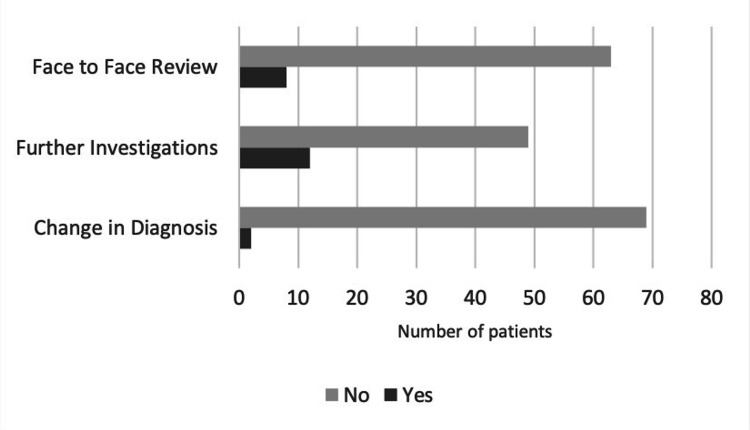
Patients needing further face-to-face review and investigations and the proportion by which the clinical diagnosis changed

Eight patients (11%) were called for a face-to-face review for clinical reassessment based on the history given and to decide if modification of the procedure or adding another procedure was needed. Twelve patients (16.9%) were sent for further investigations including radiographs and MRI scans to assess for the progression of the disease. The diagnosis was modified for two patients wherein a first MTPJ fusion was offered in place of an osteotomy and triple arthrodesis was selected instead of a limited fusion (Figure 4).

**Figure 5:**
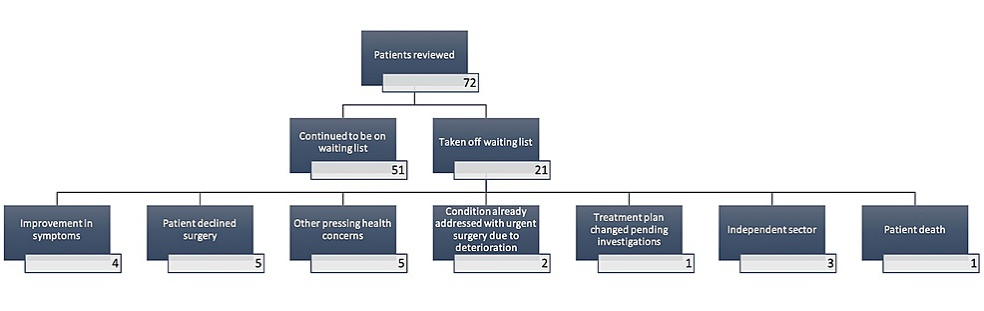
Reasons for patients being taken off the waiting list

About a third of the patients (n=21, 29.5%) were taken off the waiting list. The most common reasons to be taken off the waiting list were "other pressing health concerns" and patients declining surgery despite the symptoms being present (five patients each). Four patients showed improvement in symptoms and hence did not require an operation. Two patients had their conditions addressed already due to clinical worsening of symptoms (metalwork removal and amputation of toe) while three had their procedure done in the independent sector (the patient perspective parameters for this group were recorded as symptoms before the definitive procedure) (Figure 5).

## Discussion

Surgical waiting times can have an adverse impact on the patient’s quality of life [4]. Moreover, some patients clinically deteriorate over time when awaiting surgery [4]. At times, the surgical procedure could become more complex as a result of clinical progression [5]. As a result of the COVID-19 pandemic, there was a significant reduction in terms of compliance with the 18-week intention-to-treat target [6]. As a result of the increase in the number of patients awaiting elective surgery, it is essential to have a strategy to deal with the clinical and psychological impact of the same.

Our study shows some interesting results from the field of foot and ankle surgery. About a quarter of patients perceived a worsening quality of life and symptoms after waiting for a year for their surgeries. Some studies have reported a deterioration in the quality of life and symptoms in a third of the patients awaiting hip arthroplasty [4,7-8]. Similarly, one study has shown worsening of quality of life in about a quarter of the patients awaiting knee arthroplasty while another study showed progressive worsening of pain and disability after a wait time of more than a year [7,9].

This study also notes that about 30% of patients were taken off the waiting list for a variety of reasons: clinical and non-clinical. A similar study has noted that about 25% of patients waiting for more than six months for joint replacement surgery did not go ahead with surgeries due to several reasons such as medical delay, self-delay, alternate procedure, and death [10]. These findings underline the importance of periodic review of waitlisted patients to assess change in clinical and other priorities as more than 10% of patients in our cohort were called in for a repeat clinical examination and 16% of patients were sent for fresh imaging.

This study is unique as it is the first study that quantifies the impact of a service that has become important due to the increase in waiting times for elective surgery [11]. A possible limitation is that this study assessed the harm review service in a single orthopaedic sub-speciality in one NHS Trust only. The mental health issues experienced by the patient can be explored in further detail with the inclusion of the expertise of a psychologist. Based on the outcomes of this study, wider multi-centre audits could be conducted across different orthopaedic specialities and other specialities as well to draw broader conclusions.

## Conclusions

This study underlines the utility of a harm review service in assessing elective patients awaiting surgery. The scope of the service could be expanded to assess the impact on the patients’ mental health, quality of life, change in clinical condition, as well as the need for further assessment and investigations.
